# Dysphagia in an equine referral hospital, 182 cases

**DOI:** 10.1111/evj.14512

**Published:** 2025-05-15

**Authors:** Kevin M. Connolly, Krista Estell

**Affiliations:** ^1^ Virginia‐Maryland College of Veterinary Medicine Marion duPont Scott Equine Medical Center Leesburg Virginia USA

**Keywords:** aspiration, dysphagia, horse, pneumonia

## Abstract

**Background:**

Dysphagia describes a clinical sign of pathologies of the oral cavity, pharynx, and oesophagus that carries potentially serious consequences for horses. Given the diversity of differential diagnoses that may cause dysphagia, an understanding of the prevalence of dysphagia in hospitalised patients, the distribution of aetiologies and clinical outcomes could inform diagnosis, treatment, and prognosis.

**Objectives:**

This study aims to describe the incidence, signalment, history, aetiology, treatment, and outcome of horses presenting to a referral hospital for dysphagia.

**Study Design:**

Retrospective case series.

**Methods:**

Referral hospital cases over a 12‐year period were screened and included in the analysis for patients >6 months of age with at least one clinical sign of dysphagia and an aetiological diagnosis. Cases were partitioned into one or more aetiological categories of dysphagia (oral, pharyngeal, oesophageal, and neurogenic) based on recorded diagnosis. Treatment, survival, and resolution of dysphagia were reported.

**Results:**

Dysphagia was recorded in 1.1% of all cases. Inclusion criteria were met for 182 cases. Resolution of clinical signs for oral, pharyngeal, and oesophageal aetiologies of dysphagia was >80%, while recovery of neurogenic dysphagia was 46%. Aspiration pneumonia was a common sequela of dysphagia.

**Main Limitations:**

The retrospective design of the study was limited by the completeness of the medical record. Horses in this study population may not be representative of all dysphagia cases.

**Conclusions:**

Dysphagia is an uncommon condition in hospitalised patients. Prognosis is good for most aetiologies, but resolution of dysphagia of neurogenic origin occurs less frequently than the other causes of dysphagia.

## 1 INTRODUCTION

Feeding is a complex process involving coordination of alimentary tract structures from the rostral oral cavity to stomach with the concomitant closure of the upper respiratory tract, controlled by voluntary and reflex activity from the central and peripheral nervous system.^1^, ^2^ In humans, swallowing is a dominant process over respiration.^3^ Deglutition (swallowing) can be divided into oral, pharyngeal, and oesophageal phases; failure of one or more phases of this process can lead to *dysphagia*, described as ‘disordered eating’.^4^ Sequelae of dysphagia in horses are potentially serious health risks and include malnutrition, dehydration, electrolyte and acid/base imbalances, and aspiration pneumonia.^5^, ^6^

The broad number of structures involved in the feeding process implicates a diverse set of possible aetiologies for dysphagia; over 100 aetiologies have been described in horses.^7^ Oral dysphagia can result from pathologies that prevent prehension of feed, reduction in particle size, hydration of the food bolus, and its movement into the pharynx; these include pathologies affecting dentition, muscles of mastication and prehension, and the tongue. Pharyngeal dysphagia occurs with a failure to transport the food or liquid bolus from the caudal oropharynx to the entrance to the oesophagus, or to seal the nasopharynx and upper airway by closure of the soft palate and epiglottis. Oesophageal dysphagia occurs with the dysfunction of transport of the food bolus from the upper oesophageal sphincter to the stomach. Motor and sensory innervation of the masticatory/facial muscles, tongue, pharynx/larynx, and oesophagus, and central nervous system control of voluntary and reflex activity implicate central and peripheral neuropathies and junctionopathies as potential neurogenic causes of dysphagia.

There are no retrospective studies of dysphagia in horses, and the prevalence of dysphagia in hospitalised patients has not been reported. The distribution of aetiologies causing dysphagia and their relative outcomes is not known. Given the complexity of the feeding process and diversity of differential diagnoses that may result in dysphagia, the authors developed a clinical suspicion that resolution of dysphagia would vary between dysphagia of oral, pharyngeal, oesophageal, and neurogenic origin. To this end, we performed a retrospective review of 182 cases of dysphagia in a referral centre to describe the clinical signs, incidence, outcome, and survival of dysphagia in horses.

## 2 MATERIALS AND METHODS

### 2.1 Case selection

Case histories of all horses admitted to Virginia Tech Marion duPont Scott Equine Medical Center for evaluation between 2007 and 2019 were initially screened by keyword search for clinical signs of dysphagia: *dysphagia*, *difficulty/trouble chewing*, *difficulty/trouble swallowing*, *difficulty/trouble eating*, *salivation*, *ptyalism*, *mastication*, *prehension*, *aspiration pneumonia*, *quidding*, *dropping feed*, *coughing while eating*, *feed from nares*, *feed in trachea*, and *choke/oesophageal obstruction*. Full medical histories were evaluated by the authors, and cases >6 months of age were included in the analysis if: (a) the patient had a confirmed diagnosis of dysphagia characterised as difficult or slow mastication or deglutition, coughing while eating, nasal discharge or feed from nares while eating, dropping feed or quidding while eating; if feed was noted in the trachea during an endoscopic examination; or if oesophageal obstruction was diagnosed, and (b) diagnostic testing was performed to determine the pathology resulting in the dysphagia. Case selection was therefore a convenience sample using horses presenting to a single institution during the timeframe that hospital database searching was available.

### 2.2 Data collection

Data collected for horses with dysphagia included signalment, presenting signs, duration of signs before admission, history of prior oral/pharyngeal/oesophageal/neurological diseases, comorbidities (including whether aspiration pneumonia was diagnosed), diagnostic tests performed, diagnosis(es), treatments, duration of hospitalisation, follow‐up interval, outcome (whether signs were resolved at discharge or follow‐up), and survival was noted. Cases of dysphagia were considered resolved if the horse could prehend, masticate, and deglutinate feed without clinical signs of dysphagia. Follow‐up information was based on subsequent presentations of the horse to the hospital (for any reason), or client communications recorded in the medical record. For cases that presented for repeated episodes of dysphagia, the number of repeat presentations was recorded, but only data from the initial presentation were included. For comparison of signalment with the general hospital population, data on the sex, age, and breed of all horses admitted to the hospital during the study period were collected.

### 2.3 Categorical assignment

A dysphagia category was assigned for each diagnosed disease in the included cases. This categorisation was based on the phase of the feeding cycle the disease affected (oral, pharyngeal, or oesophageal). Neurologic or neuromuscular diseases were assigned to a fourth dysphagia category (neurogenic). Each case was then assigned to one or more dysphagia categories depending on the diagnoses at the time of hospital dismissal.

For cases in which no primary disease diagnosis was made despite investigation, the dysphagia case was categorised as idiopathic. In the cases where oesophageal obstruction was diagnosed, the case was assigned to the oesophageal dysphagia category, and any primary pathology causing the obstruction was recorded if noted in the medical record.

### 2.4 Data analysis

Descriptive statistics were determined using a commercial spreadsheet package [Microsoft Excel Version 16.84]. Signalment, history, treatment, dysphagia category, outcome (resolution of dysphagia by discharge or following hospital discharge) and survival were described.

## 3 RESULTS

### 3.1 Cases

**FIGURE 1:**
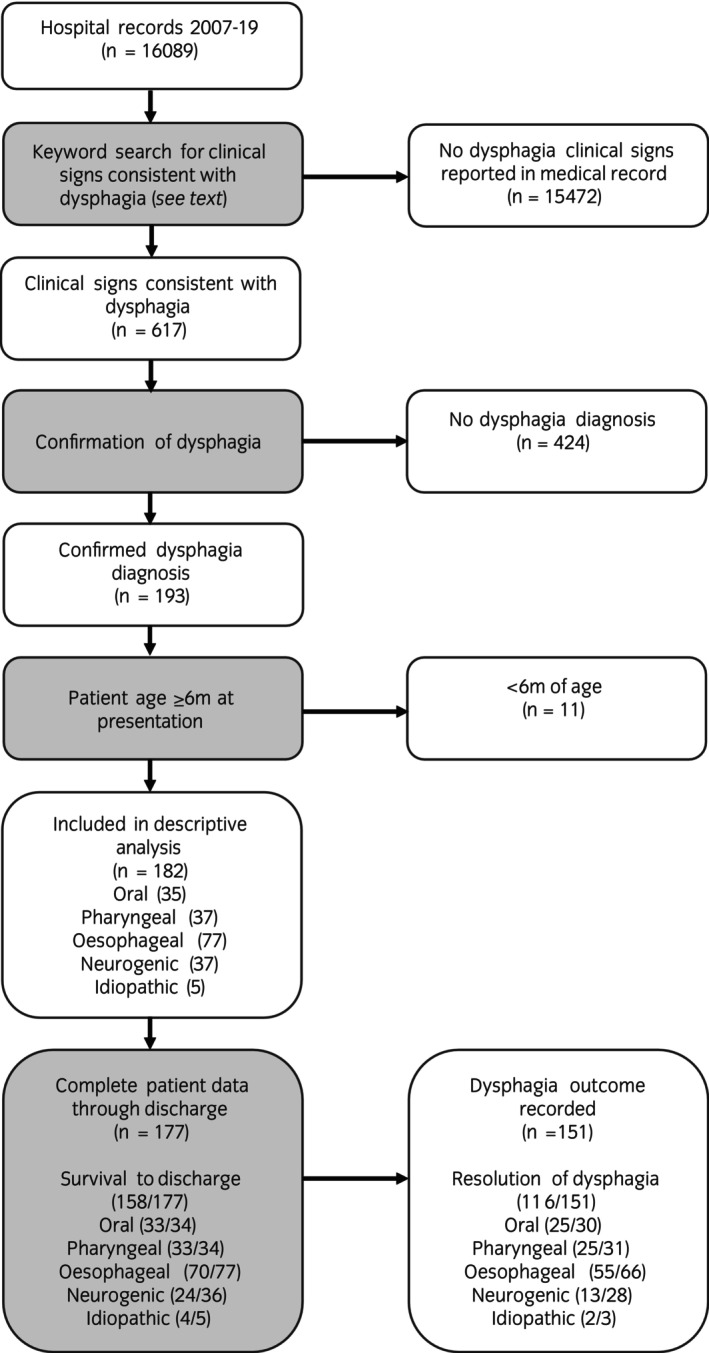
Cases selected for this study. Of the 16,089 cases during the study period, 182 cases met the inclusion criteria for descriptive analysis (confirmed dysphagia diagnosis and patient age at presentation >6 months). For cases included in descriptive statistics, the dysphagia categories are indicated (category values exceed the total number of cases as nine cases had more than one diagnosis that may have contributed to the dysphagia). Survival to discharge and resolution of dysphagia are reported for each aetiology. Complete patient data through discharge were available for 177 cases. Data on the outcome of dysphagia were available for 151 cases (this data includes non‐survivors) and were used for the descriptive statistics for resolution of dysphagia.

Records for 16,089 patients were initially screened (7941 geldings, 6918 mares, 1230 uncastrated males). Clinical signs consistent with dysphagia were reported in 617 cases; of these, 182 cases met the inclusion criteria (Figure 1). The overall prevalence of diagnosed dysphagia cases in this hospital population over the indicated period was 1.1%.

Descriptive statistics for case data are presented in Table 1. There were 108 geldings, 63 mares, and 4 stallions included in the descriptive analysis. Sex was not recorded in the medical record for 7 cases. When compared with the total hospital population, the number of geldings was slightly higher in the dysphagia group (59% of dysphagia cases vs. 49% of total hospital admissions). The number of geldings with dysphagia was higher in the three oldest age quartiles (70%, 60%, and 72% of cases in quartiles 2, 3, and 4, respectively), with mares predominating in the younger (<8‐year‐old) dysphagia cases (49% vs. 41% for geldings). The mean age at presentation was 15.2 years (range 7 months to 43 years). Thirty breeds were represented amongst the included cases, and the most represented breeds (Thoroughbred, Quarter Horse, Draught, Warmblood, and Pony breeds) were similar in distribution to the hospital population during this time period.

**TABLE 1:** Descriptive statistics for the 182 cases used in the analysis.

Descriptive case statistics (*n* = 182)	
Signalment	
Sex	Count (%)
Gelding	108/182 (59)
Mare	63/182 (35)
Stallion	4/182 (2)
Unknown	7/182 (4)
Age (years)	Median 15, Mean 15.4, Range 0.75–43
1st quartile, years	9 month to <8 years
2nd quartile, years	8 to <15 years
3rd quartile, years	15 to <22 years
4th quartile, years	22 to 43 years
Breed (30 unique)	Count (%)
Thoroughbred	57/182 (31)
Quarter Horse	24/182 (13)
Warmblood	27/182 (15)
Draught breeds	10/182 (6)
Pony breeds	15/182 (8)
Other	49/182 (27)
History	Count (%)
Referred to clinic for current oesophageal obstruction	64/182 (35)
Referred to clinic for current aspiration pneumonia	8/182 (4)
Prior dysphagia (any cause) diagnosis	65/182 (36)
Prior oesophageal obstruction	41/182 (23)
Prior pharyngeal/laryngeal disease	23/182 (13)
Prior pharyngeal/laryngeal surgery	11/182 (6)
Prior oral disease	11/182 (6)
Prior oral surgery	2/182 (1)
Prior neurological disease	3/182 (2)
Prior aspiration pneumonia	1/182 (0.5)
Dysphagia developed in hospital	9/182 (5)
Clinical signs began on day of presentation	41/182 (23)
Duration of signs before presentation (days)	Median 2, Mean 35, Range 0–730
Outcome and survival	Count (%)
Complete patient record through discharge	177/182 (97)
Outcome of dysphagia recorded	151/177 (89)
Euthanised/died in hospital	19/177 (11)
Dysphagia resolved	116/151 (77)
Dysphagia resolved before discharge	88/151 (58)
Re‐presented with dysphagia	13/151 (9)
Follow‐up period (days)	Median 7, Mean 203, Range 1–2480
Hospitalisation (days)	Median 3, Mean 6, Range 0–52
Hospitalisation cost (dollars)	Median 2239, Mean 3334, Range 77–19,945

*Note:* For each category, cases are expressed as proportions and percent of cases (in parentheses). For continuous variables (age, duration of clinical signs, follow‐up period, hospitalisation duration, and cost), mean and median values, and range are indicated.

### 3.2 Historical data and clinical signs

Historical data are presented in Table 1. Sixty‐five of 182 cases presented with a history of a prior diagnosis of dysphagia. Putative risk factors for dysphagia (prior histories of oral, pharyngeal/laryngeal, or neurogenic diseases, or prior history of oesophageal obstruction) were indicated for 6%, 13%, 2%, and 23% of cases, respectively (Table 1). Sixty‐four of 182 cases were referred for acute oesophageal obstruction, and 8 of these cases had a tentative diagnosis of aspiration pneumonia made before referral. In 5% of cases (9/182), dysphagia had developed in hospital. In 23% of cases (41/182), referral for evaluation was made on the same day that clinical signs of dysphagia were observed. For the remainder of cases, the median number of days between the emergence of signs of dysphagia and hospital admission was 2 days.

**FIGURE 2:**
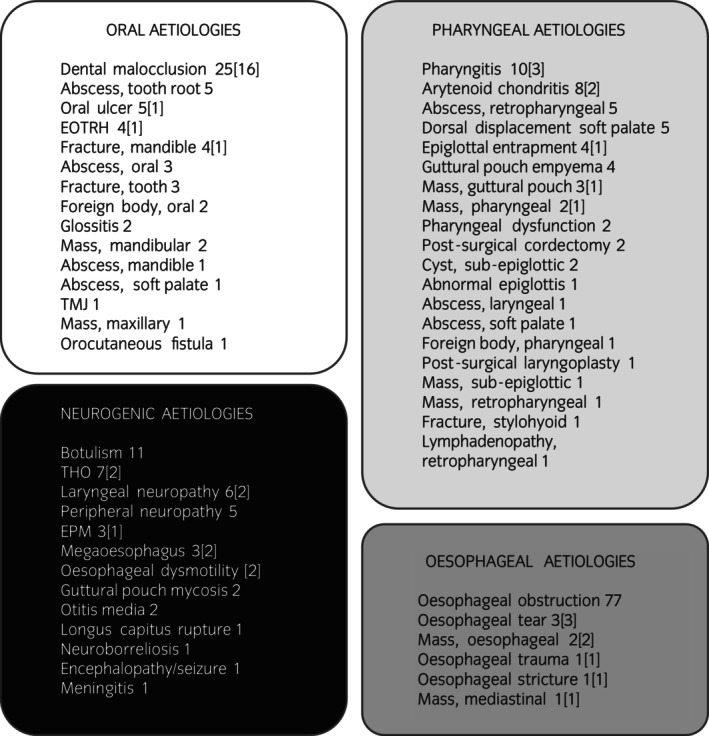
Categories of dysphagia and corresponding diagnoses. Each disease resulting in dysphagia was assigned to a dysphagia category (oral, pharyngeal, oesophageal, and neurogenic). The total number of cases in this analysis for each pathology is indicated. Numbers in brackets indicate the number of cases in which the pathology was diagnosed as a cause of oesophageal obstruction. Note that the total of all disease diagnoses exceeds the total number of cases, as some cases had more than one diagnosis for dysphagia. EOTRH, Equine Odontoclastic Tooth Resorption and Hypercementosis; THO, temporohyoid osteoarthropathy.

**FIGURE 3:**
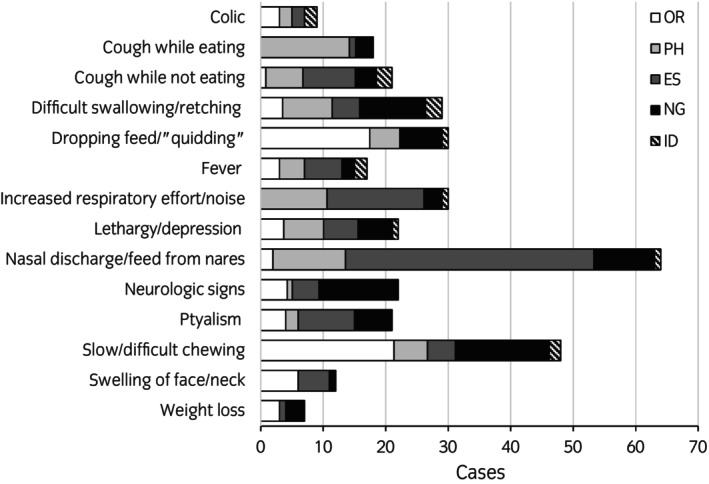
Clinical signs recorded for dysphagia cases. Bars indicate the total number of cases in which the clinical signs were noted. Divisions within bars indicate the percentages of cases in which the clinical sign was present for each of the dysphagia aetiological categories. ID, idiopathic dysphagia; NG, neurogenic dysphagia; OR, oral dysphagia; OS, oesophageal dysphagia; PH, pharyngeal dysphagia.

Categories of dysphagia (oral, pharyngeal, oesophageal, and neurogenic) and corresponding diagnoses are reported in Figure 2. Common clinical signs (signs observed in more than one dysphagia case) are presented in Figure 3. The most common clinical signs were nasal discharge (64/182 cases; 35%) and difficulties with mastication (48/182 cases; 26%). Perhaps not surprisingly, most of the common clinical signs varied in frequency in respect to aetiological categories of dysphagia. Nasal discharge was more commonly observed in patients with oesophageal dysphagia (41/64; 64%), while slow mastication/dropping feed was more commonly seen with oral dysphagia (18/30; 60%) and coughing while eating was more common in pharyngeal dysphagia patients (15/18; 83%).

### 3.3 Diagnoses and aetiological categorisation

Given the number of differential diagnoses for dysphagia, a wide range of diagnostic tests was utilised (Figure S1). Endoscopy was the most common diagnostic technique (144/182 cases, 79%), followed by ultrasound (87/182, 48%), radiography (56/182, 31%) and oral examination (56/182, 31%). Complete blood count and chemistry were available for 68% and 53% of cases, respectively.

**FIGURE 4:**
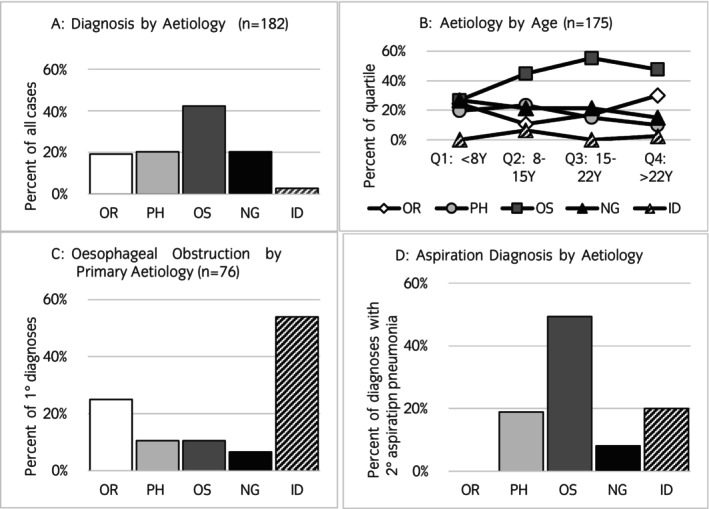
(A) Distribution of dysphagia diagnoses across the aetiological categories. Diagnoses of the cases are based on the aetiological category of dysphagia. Bars indicate the percentage of all dysphagia cases that were assigned to each aetiological category (ID, idiopathic dysphagia; NG, neurogenic dysphagia; OR, oral dysphagia; OS, oesophageal dysphagia; PH, pharyngeal dysphagia); (B) Distribution of dysphagia diagnoses across the aetiological categories by patient age. For each age quartile, the distribution of diagnoses by aetiological category is indicated by the markers as a percentage of all diagnoses in that age quartile; (C) Primary causes of oesophageal obstruction based on aetiology. For each aetiological category, the bar represents the percentage of all oesophageal obstruction cases that were assessed to be secondary to dysfunction in that aetiological category; (D) Diagnoses of secondary aspiration pneumonia for the aetiological category of dysphagia. Each bar represents the percentage of all cases within that aetiological category that were diagnosed with secondary aspiration pneumonia.

The distribution of dysphagia diagnoses for each aetiological category is presented in Figure 4A. Oesophageal dysphagia was most observed in this population (77/182; 42%), while oral, pharyngeal, and neurogenic dysphagia was diagnosed in 19%, 20%, and 20% of cases, respectively. Only 3% (5/182) of cases did not have a definitive diagnosis and were categorised as idiopathic. Nine cases were diagnosed with >1 primary disease for which dysphagia was a potential clinical sign (3 horses with simultaneous pharyngeal/neurogenic diseases, 2 with simultaneous oral/pharyngeal diseases, 3 with simultaneous oral/neurogenic diseases, and 1 with simultaneous oesophageal/neurogenic diseases).

The distribution of aetiologies of dysphagia differed by age (Figure 4B). Neurogenic aetiologies were most common in younger patients, oesophageal aetiologies were most common in middle‐aged patients, and oral aetiologies were most common in older patients. Oesophageal obstruction was the most common diagnosis (all 77 cases of oesophageal dysphagia were associated with obstruction); in 36 of these cases, one or more primary pathologies could be determined as contributing to oesophageal obstruction (Figure 4C). The most common cause of oral dysphagia was attributed to dental disease (malocclusion, tooth root abscesses, and EOTRH), though other oral pathologies were recorded (Figure 2).

Bronchopneumonia secondary to aspiration (aspiration pneumonia) was a common sequela of dysphagia and was observed in 27% (49/182) of cases. The prevalence of aspiration pneumonia for cases of each aetiological category is presented in Figure 4D. Aspiration pneumonia was most frequently diagnosed in oesophageal dysphagia cases; 49% of these cases had aspiration pneumonia secondary to dysphagia. Of the 19 cases that did not survive to discharge, 8 (42%) died or were euthanised due to complications arising from aspiration pneumonia.

### 3.4 Treatment and outcome

Treatments varied by primary disease diagnosis, and many were specific to the primary disease process (e.g., botulism antitoxin, dental equilibration, and anticholinergics). The most common therapies used across all aetiological causes of dysphagia were antibiotics (65% of cases), anti‐inflammatories (NSAIDs, systemic and topical glucocorticoids, DMSO; 69% of cases), and IV fluid therapy (31% of cases). In 110/119 cases where antibiotics were prescribed, bacterial infection was suspected by thoracic imaging and/or complete blood count (including all cases where 3rd generation cephalosporins or enrofloxacin was administered).

Gastroprotectants (omeprazole, sucralfate, and ranitidine) were administered in 20% of cases and were used more frequently for oral and oesophageal aetiologies of dysphagia. Therapeutic feeding was used in 45% of dysphagia cases and included special diets (36%), parenteral nutrition (6%), and feeding via nasogastric tube (3%).

A medical record describing survival to discharge was documented in 177/182 cases (97%) and is outlined in Figure 1. Survival for horses with dysphagia was generally positive, with 158/177 (89%) of horses surviving to discharge. Data regarding the outcome of the dysphagia (resolved vs. unresolved) was recorded in the medical record for 151/177 cases (85%). Resolution of dysphagia (disappearance of clinical signs) was reported for 116/151 (77%) of cases; 88 of these 116 cases (76%) resolved before discharge. Resolution of dysphagia was less frequent for horses with neurogenic dysphagia, with only 46% recovering, while horses with oral, pharyngeal, and oesophageal causes of dysphagia recovered at rates of 83%, 81%, and 83%, respectively.

Nineteen horses with dysphagia (11%) did not survive to discharge (Figure 1). Of these 19 horses, 10 were euthanised due to neurological diseases with perceived poor prognoses (6 botulism, 1 peripheral neuropathy, 1 megaoesophagus, 1 ruptured longus capitus muscle, and 1 meningitis), and 1 was euthanised due to a complicated oesophageal tear. Eight cases were euthanised due to complications from aspiration pneumonia. Non‐surviving horses with aspiration pneumonia were younger than survivors (mean age 11 vs. 16 years) and had no additional comorbidities at presentation recorded in the medical record. Survival was generally good for oral, pharyngeal, and oesophageal causes of dysphagia with survival to discharge rates of 97%, 97%, and 91%, respectively. A primary diagnosis for dysphagia was not achieved in five cases, and they were assigned to the ‘idiopathic’ category. Four of five horses (80%) with idiopathic dysphagia survived to discharge.

## 4 DISCUSSION

This study represents the first retrospective analysis of dysphagia cases in a referral centre. Dysphagia, a dysfunction of prehension, mastication, and/or deglutition, was uncommonly diagnosed in our hospital population (1.1% of all hospital cases during a 12‐year period), despite the prevalence of some of the inciting causes (dental malocclusion, pharyngeal/laryngeal diseases) in the hospital records. While there are several differential diagnoses for abnormalities in the feeding process, a definitive aetiological diagnosis of a primary disease causing this clinical sign in our patient population was common, with 177/182 cases recording a determinative cause that could be classified as oral, pharyngeal, oesophageal, or neurogenic dysphagia. This implies a high probability for obtaining an aetiological diagnosis for dysphagia with investigation. Oesophageal dysphagia (specifically oesophageal obstruction) was the most common diagnosis in our data set. While oesophageal obstruction is itself a diagnosis, it can be secondary to additional pathology. In 46% of oesophageal obstruction cases in our study, a primary cause attributed to oral, pharyngeal, or neurogenic pathology was identified and suggests the importance of primary diagnosis for an appropriate therapy plan. Dental abnormalities were the most common suspected cause of oesophageal obstruction, a finding seen in other cross‐sectional studies of oesophageal obstruction cases.^8^

Signalment may affect dysphagia incidence. There were slightly more geldings in our dysphagia cases when compared with the hospital population; 59% of dysphagia cases were geldings while only 49% of total hospital admissions were geldings, and this increased frequency of geldings amongst dysphagia cases was observed across the three oldest age quartiles. Resolution of dysphagia following treatment did not differ between patient sex. Categorical diagnoses appeared to differ between horses by age; while similar in frequency in the youngest quartile, oral and oesophageal causes of dysphagia tended to increase with patient age, while neurogenic and pharyngeal causes tended to decrease. These trends may not be unexpected given the age‐related incidence of some of the underlying pathologies within each of these categories (Figure 2).^9^ The most common oral and oesophageal pathologies in our data (dental malocclusion and oesophageal obstruction) are conditions with generally good prognoses, while the most frequent neurogenic causes (botulism and peripheral neuropathies) carry poorer prognoses. This is reflected by the survival rate; of the 19 dysphagic horses that died or were euthanised, 10 had neurogenic dysphagia.

More than 80% of cases with oral, pharyngeal, and oesophageal dysphagia diagnoses resolved with treatment. Resolution of clinical signs in dysphagia cases of neurogenic origin was lower compared with other aetiologies, and the majority of non‐surviving cases were diagnosed with a neurogenic aetiology as the cause for the observed dysphagia. This finding is not surprising given the nature of the pathologies associated with the dysphagia in our study (Figure 2). Several of these pathologies can carry a guarded prognosis (botulism) or the potential for persistent neurological abnormalities (temporohyoid osteoarthropathy,^10^ Equine Protozoal Myeloencephalitis,^11^ peripheral neuropathy). Botulism was the most common neurogenic cause of dysphagia in our study (11 cases); resolution of dysphagia was reported in 4 cases, while 6 cases did not survive to discharge.

Aspiration pneumonia was the most reported complication of dysphagia and was diagnosed in 27% of dysphagia cases. While most commonly occurring secondary to oesophageal dysphagia (nearly half of cases), aspiration pneumonia was diagnosed secondary to pharyngeal and neurogenic dysphagia as well. Aspiration pneumonia incidence amongst cases with oesophageal obstruction diagnosis was similar to that reported previously.^12^ Nearly half of non‐surviving cases had a secondary aspiration pneumonia diagnosis, and in these cases, pneumonia complications were indicated as the reason for euthanasia. Given the prevalence of aspiration pneumonia as a common sequela for all causes of dysphagia and its potentially poor prognosis, the data suggest rigorous diagnosis and appropriate treatment may be warranted in dysphagia cases regardless of suspected aetiology.^12^

The dataset size was not sufficiently large to fully assess the effect of all individual treatment(s) on outcomes. Antibiotics, anti‐inflammatories, and therapeutic nutrition were the most common modalities used routinely for the treatment of dysphagia across all aetiological categories. Antimicrobials were administered to over half of all dysphagia cases and were chosen at the discretion of the attending veterinarian. Justification of antibiotic use was not always present in the medical record; however, diagnostic results consistent with infection were obtained before antibiotic therapy was initiated in most instances. It is important to note that in some cases, enrofloxacin and ceftiofur, a third‐generation cephalosporin, were used in the treatment of aspiration pneumonia. Though justification was not present in the medical record, in some instances, horses were azotemic and it is surmised that enrofloxacin was used as an alternative to aminoglycosides for the treatment of gram‐negative bacteria. A more appropriate combination of first‐line antimicrobials such as sulfonamides, often with metronidazole, or penicillin and an aminoglycoside was administered in the majority of cases, and the authors recommend responsible, judicious use of antimicrobials. The use of high‐priority critically important antimicrobials should be reserved for cases that have bacterial cultures demonstrating that there are no alternatives available and that the bacteria are susceptible to these antimicrobials at concentrations that are achievable in horses.

Limitations of this study are its retrospective design and therefore the reliability of the historical and clinical information, and the completeness of the medical records from which data were gathered. Additionally, there is a potential for bias in the diagnosis of the primary pathology which resulted in dysphagia as a clinical sign. As this study was performed using a referral hospital patient population, it may not be representative of the total population of horses diagnosed with any cause of dysphagia in primary practice. Outcomes of these cases were dependent on the available diagnostic and treatment modalities and may not be broadly applicable. The classification scheme used to partition cases based on the phase of feeding they affect (oral, pharyngeal, oesophageal, and neurogenic) was a potential source of observer bias. Some diagnoses (recurrent laryngeal neuropathy, peripheral neuropathies of facial and masticatory muscles, guttural pouch diseases) can be classified in more than one aetiological category, but a single classification was used to clarify comparison. As the incidence of dysphagia has not been reported previously in horses, a power analysis was not performed before initiating the study; rather, medical records were searched over 12 years, which represented the extent of the medical records database at our hospital. Finally, dysphagia is not a disease, but a clinical sign secondary to another pathology. Whether it was independent from or secondary to the recorded diagnoses could not be confirmed, although the resolution of dysphagia following treatment of the primary disease in our data suggests the latter.

## FUNDING INFORMATION

Funding for publication of this article was provided by the Virginia Tech Marion duPont Scott Equine Medical Center Foundation.

## CONFLICT OF INTEREST STATEMENT

The authors have declared no conflicting interests.

## AUTHOR CONTRIBUTIONS

**Kevin M. Connolly:** Conceptualization; investigation; funding acquisition; writing – original draft; methodology; validation; visualization; writing – review and editing. **Krista Estell:** Conceptualization; investigation; methodology; validation; visualization; writing – review and editing.

## DATA INTEGRITY STATEMENT

Kevin M. Connolly gathered, compiled, and analysed the data. Krista Estell reviewed the data in its entirety.

## ETHICAL ANIMAL RESEARCH

Research ethics committee oversight not required by this journal: retrospective study of clinical records.

## INFORMED CONSENT

Owners gave general consent for use of medical records for research.

## ANTIMICROBIAL STEWARDSHIP POLICY

Not applicable.

## Supporting information


**Figure S1.** Range of diagnostic tests utilised.

## Data Availability

Data underlying this manuscript are made accessible through the Virginia Tech Data Repository at https://doi.org/10.7294/28938926.
