# Supplementary figures and images for: Dysphagia in an equine referral hospital, 182 cases
Source: Equine Vet J. 2025 May 15;58(1):134–42. doi: 10.1111/evj.14512 (PMC12699097; doi:10.1111/evj.14512)

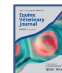

**Figure S1:** Range of diagnostic tests utilised.

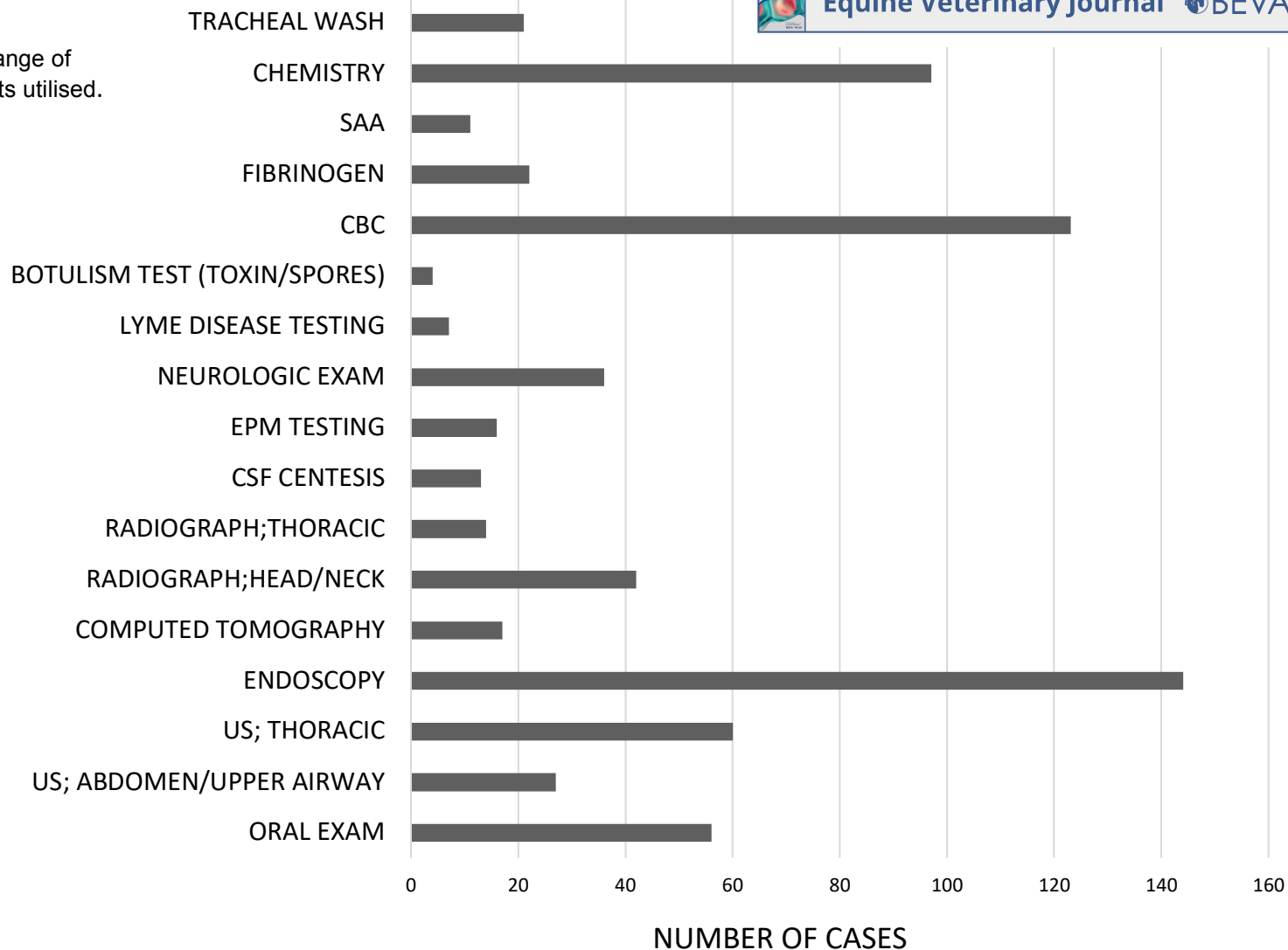

Supplement: Supplementary file 1 — Figure S1. Range of diagnostic tests utilised. [file EVJ-58-134-s001.pdf]
